# Growth and survival among Hawaiian corals outplanted from tanks to an ocean nursery are driven by individual genotype and species differences rather than preconditioning to thermal stress

**DOI:** 10.7717/peerj.13112

**Published:** 2022-03-23

**Authors:** E. Michael Henley, Jessica Bouwmeester, Christopher P. Jury, Robert J. Toonen, Mariko Quinn, Claire V.A. Lager, Mary Hagedorn

**Affiliations:** 1 Smithsonian Conservation Biology Institute, Smithsonian Institution, Front Royal, Virginia, United States; 2 Hawai‘i Institute of Marine Biology, University of Hawai‘i at Mānoa, Kāne‘ohe, Hawai‘i, United States

**Keywords:** Resilience, Coral nursery, Assisted evolution, Coral tree, Coral farm, Adaptive potential, Acclimatization, Bleaching, Acropora, Reef restoration

## Abstract

The drastic decline in coral coverage has stimulated an interest in reef restoration, and various iterations of coral nurseries have been used to augment restoration strategies. Here we examine the growth of two species of Hawaiian *Montipora* that were maintained in mesocosms under either ambient or warmed annual bleaching conditions for two consecutive years prior to outplanting to determine whether preconditioning aided coral restoration efforts. Using coral trees to create a nearby ocean nursery, we examined whether: (1) previous *ex situ* mesocosm growth would mirror *in situ* coral tree nursery growth; and (2) thermal *ex situ* stress-hardening would predict future success during natural warming events *in situ* for corals moved from tanks to trees. For *Montipora capitata*, we found that variation in growth was explained primarily by genotype; growth rates in the mesocosms were similar to those *in situ*, irrespective of preconditioning. Variation in *M. flabellata* growth, however, was explained by both genotype and culture method such that an individual *M. flabellata* colony that grew well in the tanks did not necessarily perform as well on the coral trees. For both species, previous exposure to elevated temperatures in the mesocosms provided no benefit to either growth or survival during a warming event in the coral tree nursery compared to those grown in ambient temperatures. Overall, *M. capitata* performed better in the tree nursery with higher net growth, lower mortality, and was subject to less predation than *M. flabellata*. Our results show little benefit of the additional cost and time of stress-hardening these corals prior to outplanting because it is unlikely to aid resilience to future warming events. These results also suggest that selecting corals for restoration based on long-term genotype growth performance may be more effective for optimal outcomes but should be weighed against other factors, such as coral morphology, *in situ* nursery method, location, and other characteristics.

## Introduction

Coral reefs around the world are in decline from combined global and local environmental stressors such as climate change, nutrient pollution and runoff, sedimentation, and destructive fishing practices (Fabricius, 2005; Hoegh-Guldberg et al., 2007; Hughes et al., 2017; Jackson et al., 2001; Zaneveld et al., 2016). When one or more of these stressors are alleviated, it is possible that an area of degraded reef might be a candidate for restoration (Anthony, 2016; Bahr, Jokiel & Toonen, 2015; Jury & Toonen, 2019; van Oppen et al., 2017). Classic coral reef restoration projects have typically used ‘fragments of opportunity’ (broken coral fragments collected after a storm, boat grounding, or other mechanical disturbance) or other sources of asexual fragments, which are then grown in an underwater nursery (‘coral garden’) and later transplanted to the reef (Epstein, Bak & Rinkevich, 2003; Rinkevich, 2000; Rinkevich, 2014; Shafir, Van Rijn & Rinkevich, 2006). Corals in these *in situ* nurseries have been grown on cinder blocks, wire or PVC table structures, rope lines, and floating platforms (Boström-Einarsson et al., 2020; Young, Schopmeyer & Lirman, 2012). More recently, restoration strategies have expanded to include using planulae and/or settled sexual recruits to seed the reef (Boström-Einarsson et al., 2020; Chamberland et al., 2015; dela Cruz & Harrison, 2017) or small ‘microfragments’ attached to substrate, grown *ex situ* in holding tanks, and transplanted to the reef when those individuals have fused into a larger colony (Forsman et al., 2015; Page, Muller & Vaughan, 2018). However, because holding and caring for a coral in captivity for long periods of time increases the cost-per-unit and reduces the scope of the restoration effort, high financial costs and the small scale of restoration programs can limit effectiveness at the ecological scale (Guest et al., 2014; Young, Schopmeyer & Lirman, 2012).

Coral nursery practitioners, particularly those in Florida and the Caribbean, continue to develop techniques that show promise for lower cost, large scale reef restoration projects that can be exported to other locations around the world. A relatively simple vertical PVC and horizontal fiberglass rod structure secured to the ocean floor–dubbed ‘coral trees’–allows corals to be grown *in situ via* suspension by a monofilament line in the water column. This method reduces the burden of threats and potential sources of mortality characteristic of typical coral benthic habitat (sedimentation, algal competition, and even some predation) that plague early sexual recruits and small asexual fragments (Nedimyer, Gaines & Roach, 2011). While coral trees have demonstrated success in a few locations, the technique needs to be examined in other areas, for not all reef environments are the same, and potential modifications might need to be explored and tested.

Often larger sized coral colonies have an increased chance of survival than smaller counterparts (Becker & Mueller, 2001; Pausch et al., 2015). For large scale reef restoration projects with multi-tiered components, sexual recruits or small fragments that have been settled or grown *ex situ* in holding tanks and stabilized after a short period of time might benefit from a grow-out period in an *in situ* nursery (*e.g*., on coral trees) to further increase in size prior to outplanting, potentially increasing survivorship while keeping costs manageable (Guest et al., 2014; Lirman & Schopmeyer, 2016). It has been demonstrated in both *ex situ* and *in situ* culture methods that some individuals of a species tend to calcify faster than conspecifics, suggesting that genotype plays an important role in growth performance (Bahr et al., 2020; Drury, Manzello & Lirman, 2017; Jury & Toonen, 2019; Lohr & Patterson, 2017; O’Donnell et al., 2017). What is unclear is whether the growth performance from individuals in the *ex situ* nursery will indicate future success when transferred to the *in situ* ocean nursery and, eventually, to the reef (Edmunds & Putnam, 2020).

Further complicating restoration initiatives, rising ocean temperatures are a recurring threat to the persistence of reefs (Hughes et al., 2018). Repeated bleaching events increase the susceptibility of some species to future warming (Grottoli et al., 2014), but there is some evidence for potential acclimatization or adaptation to increasing temperatures if corals survive a warming event (Bahr, Rodgers & Jokiel, 2017; Coles et al., 2018; Guest et al., 2012; Jury & Toonen, 2019; Maynard et al., 2008). Trying to draw upon and propagate this variation, assorted intervention strategies have been proposed to augment restoration programs (van Oppen et al., 2017). Some methods include selective breeding (Chan et al., 2018; Quigley, Bay & van Oppen, 2020), assisted gene flow (Hagedorn et al., 2021), microbiome manipulation (Damjanovic et al., 2019; Riegl et al., 2011; Rosado et al., 2019), and preconditioning (Morikawa & Palumbi, 2019; Putnam & Gates, 2015) which are designed to increase tolerance to environmental pressures, such as increasing thermal stress (Kleypas et al., 2021). The practicality of stress hardening corals to adapt to future climate conditions is an active area of interest within the field of conservation and restoration.

In the present study, we sought to examine if the growth performance differences identified among coral genotypes cultured in mesocosms were retained when they were transferred back to the ocean during a transition grow-out period on coral trees and if thermal preconditioning while in the mesocosms affected their subsequent responses during a natural warming event. Found in a diverse array of Hawaiian reef habitats, *Montipora capitata* is a dominant species that has both branching and plating morphologies, whereas *M. flabellata* displays an encrusting growth form that is found primarily in shallow areas of high wave action and irradiance (Fenner, 2005; Hunter & Evans, 1995; Rodgers et al., 2015). Using small fragments of *M. capitata* and *M. flabellata* from a prior 2-year mesocosm study (Bahr et al., 2020; McLachlan et al., 2022; Timmers et al., 2021), we tracked the growth of corals on the trees through an additional year and compared that to their previous performance in the mesocosm system. Additionally, because subsets of corals were also grown under present-day average and high temperature conditions as part of the original study, we investigated what effect, if any, the exposure to heat stress had on growth rate and survival of corals from the high temperature tanks compared to those from the ambient system when exposed to a natural warming event while growing on the coral trees. Finally, we compared the overall general performance on the coral trees of the two species.

## Methods

### Previous experiment and coral history

**Figure 1:**
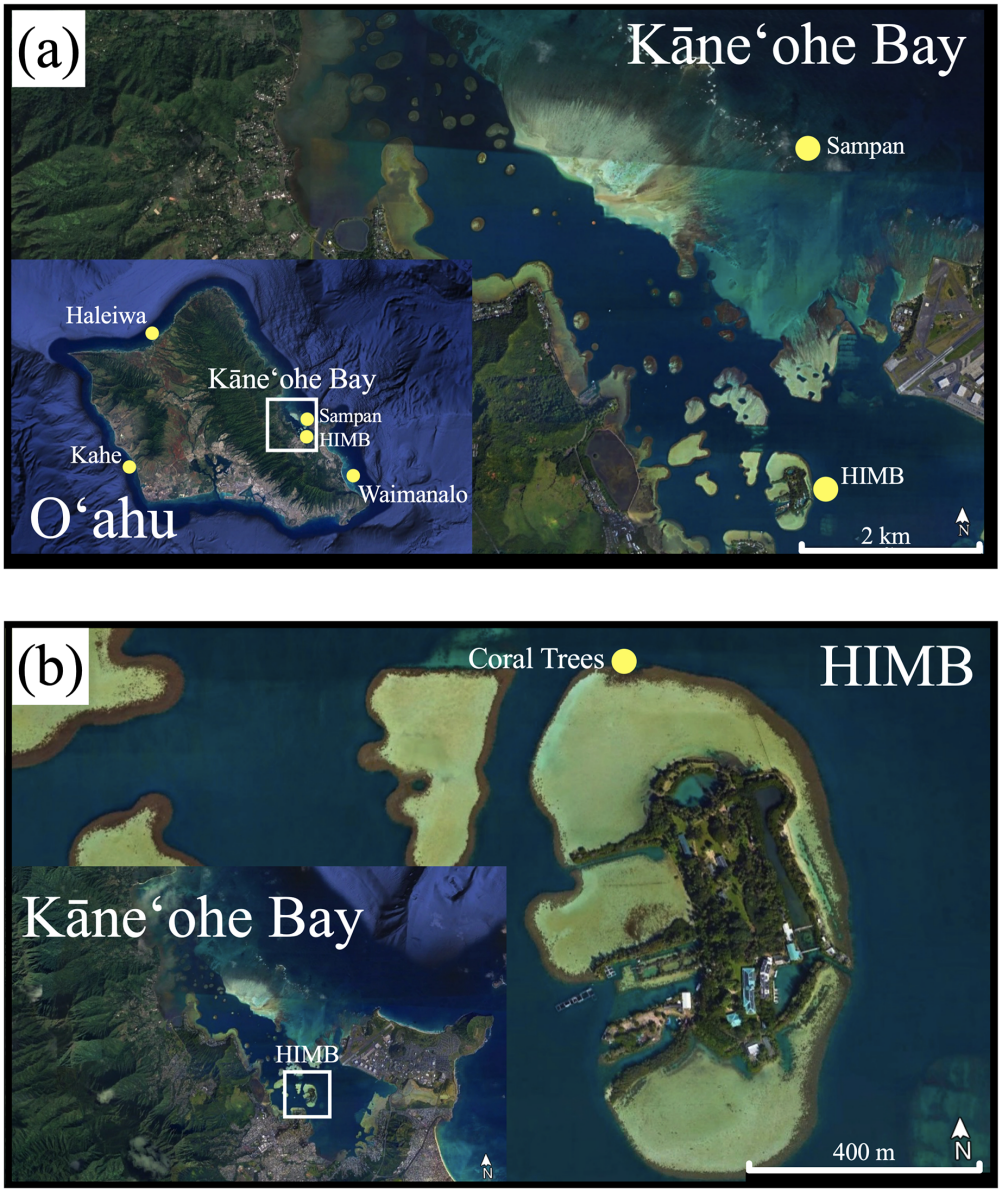
Original coral collection and tree nursery. (A) Map of O‘ahu with original collection sites of corals grown in mesocosms for the original study. (B) Coral tree nursery location on the north side of the HIMB reef in Kāne‘ohe Bay. (*Google Earth Pro* v. 7.3.3.7786, www.earth.google.com).

The corals used in this nursery experiment were obtained from a concluding study that were grown in mesocosms for approximately two years at the Hawai‘i Institute of Marine Biology in Kāne‘ohe Bay, Hawai‘i. Corals from the original *ex situ* mesocosm studies were collected from different locations around O‘ahu (Haleiwa, the reef around HIMB, Kahe, Sampan Channel, and Waimānalo; Fig. 1A) and housed in flow-through seawater tanks at HIMB. Over the 2-year mesocosm study, many of these corals grew into larger colonies from which fragments for the present study were derived (described in Bahr et al., 2020; McLachlan et al., 2022; Timmers et al., 2021).

Briefly, each mesocosm from the previous experiment experienced natural daily and seasonal fluctuations in light, seawater temperature, and carbonate chemistry with the temperature treatments set to either the present-day 2-week average of O‘ahu (hereafter referred to as ambient) or +2 °C elevated seawater temperatures (high temperature) that simulated future ocean conditions with approximately 24 degree heating weeks per year of bleaching stress (McLachlan et al., 2022; Timmers et al., 2021). Buoyant weight converted to dry weight was used to determine growth (Jokiel, Maragos & Franzisket, 1978). Corals were originally collected under Special Activity Permit numbers SAP 2015-17 and SAP 2016-69, and the coral tree nursery experiment was conducted under Site Plan Approval SPA OA-17-45. All permits were issued by the State of Hawai‘i Department of Land and Natural Resources.

### Colony fragmentation and coral tree deployment

**Figure 2:**
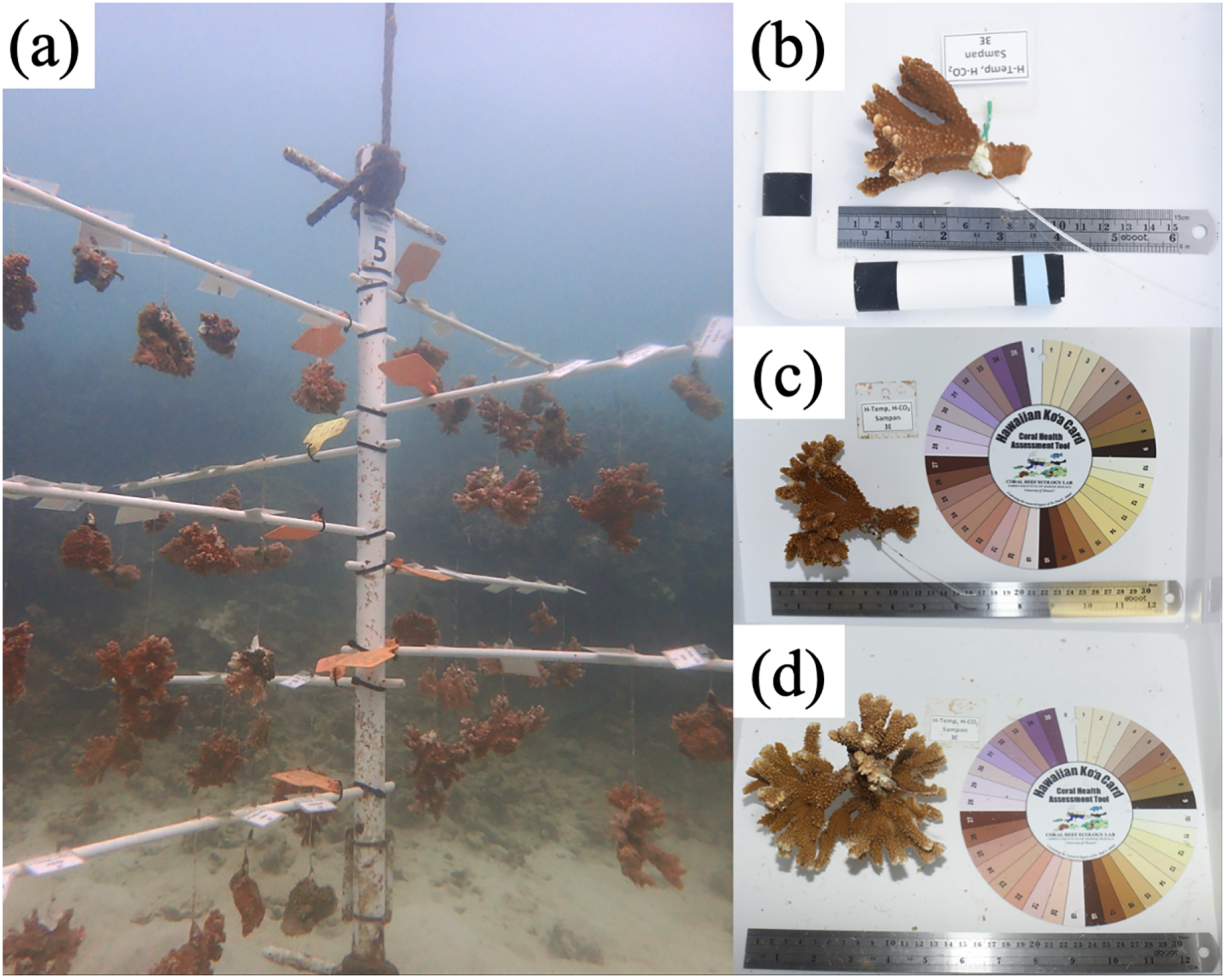
Coral tree and fragment growth. (A) Coral tree at HIMB with tree number, branch number, coral ramet identification, and suspended corals characteristic of the method. Due to the shallow depth (4 m), the trees were shorter than typical coral trees anchored in deeper water and the number of corals per branch doubled from three to six. (B) Initial size of *M. capitata* ramet beginning in Jan/Feb 2019, (C) mid-point size Aug/Sept 2019, and (D) final size Feb/Mar 2020 of the same ramet. Note that the coral more than doubled in size as did its weight. Photo credits: author.

In total, 10 coral trees were secured near HIMB in Kāne‘ohe Bay (21°43′769″N, 157°78′962″W), anchored at a depth of 4 m, and were installed in a line along the reef with alternating species per tree: *M. capitata* trees 1, 3, 5, 7, 9; *M. flabellata* trees 2, 4, 6, 8, 10 (Figs. 1B, 2A). For both species, one colony of each genet (genotype) remaining in both ambient and higher temperature treatments in the original mesocosm study (McLachlan et al., 2022; Timmers et al., 2021) was selected to fragment into ten smaller ramets (replicate fragments) to be deployed to the coral trees. Care was taken to make ramets from each colony approximately the same size, with each initially no larger than ~7 × 10 cm. Each ramet was tagged using the same colony site location, number, and treatment from the preceding study with an additional identifier applied to track each ramet. To be comparable to the original mesocosm study, corals were then weighed using buoyant weight (Mettler PM2000 scale; Mettler-Toledo, Columbus, OH, USA) that was later converted to dry weight (Jokiel, Maragos & Franzisket, 1978) and photographed (Nikon D810 with 50 mm lens; Nikon Inc., Melville, NY, USA) (Fig. 2B).

**Figure 3:**
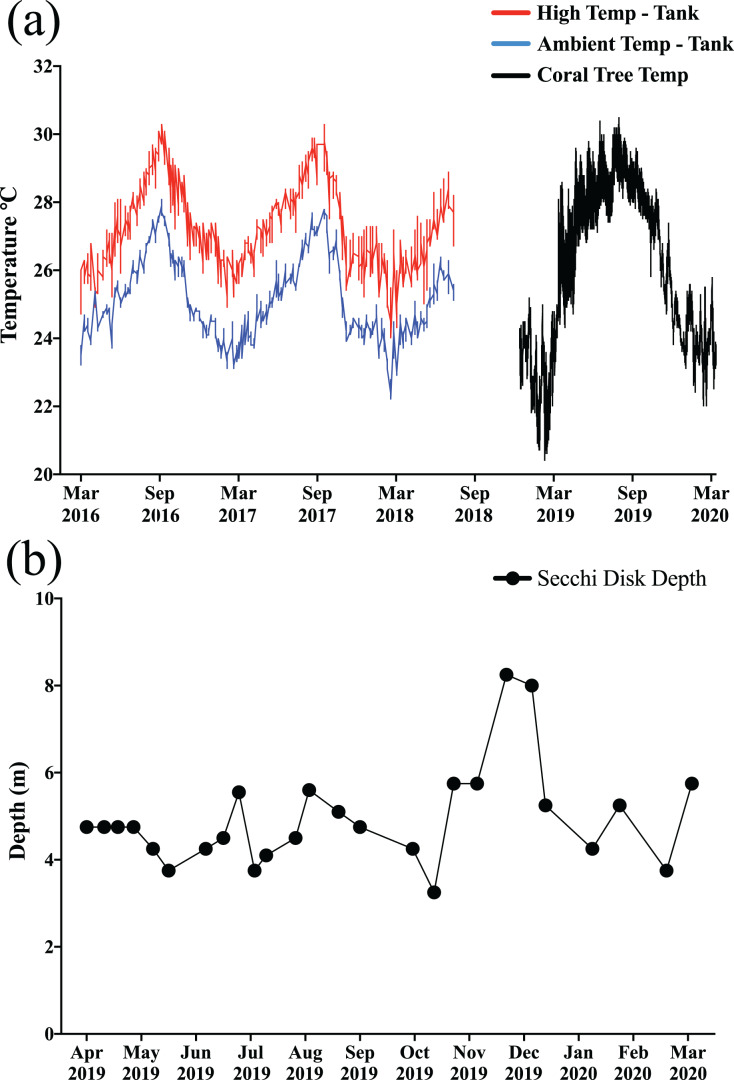
Temperature and water clarity. (A) The colored lines on the left side of the graph the portray the high (red) and ambient (blue) temperature profiles for the *ex situ* mesocosms (prior to these experiments), and the *in situ* temperatures (black) for the coral trees are on the right side of the graph. (B) Secchi disk turbidity readings near coral trees. The *in situ* exposure covered the entire range of temperatures that the corals experienced in their *ex situ* mesocosms prior to being transplanted onto the trees. Moreover, except for a few months during the winter, all of the trees experienced a relatively constant, but low light regime, due to turbidity in the bay.

After weighing and photographing, ramets from each species were haphazardly selected and secured to coral tree branches (fiberglass rods) with monofilament line and crimp *via* the hanging arrangement per methods used by Nedimyer, Gaines & Roach (2011), resulting in a haphazard mixture of corals from each temperature treatment and mesocosm on each tree. In addition to each individual coral having a tag, each branch on each tree was also labeled, and the order of corals on each branch was recorded to ensure proper identification in the event that some tags were lost. Ramets of *M. capitata* (*n* = 216) were secured to the trees in late January through early February 2019 while those of *M. flabellata* (*n* = 290) were deployed the following month. The trees were inspected one to two times per month, and the few times fragments had fallen they were placed back on the tree. HOBO temperature loggers (HOBO Pendant© Temperature 64K Data Logger; Onset, Bourne, MA, USA) were used to record temperature, and Secchi disk measurements (Preisendorfer, 1986) were taken at least two to three times per month to monitor turbidity and water clarity (Figs. 3A and 3B).

At six months post deployment–throughout August and early September 2019–branches from individual trees were brought into and temporarily housed in tanks with flow-through seawater and 70% shade cloth. Corals were removed from the branches, photographed, and buoyant weights recorded. As part of the visual assessment, data were also recorded and estimated for tissue loss (partial/total mortality, if any), any paling or bleaching, and if there were signs of significant predation, approximately how much of the ramet was lost or if it had completely disappeared from the coral tree. Corals were redeployed to their respective location on each tree within four days of removal (Fig. 2C).

For many corals–particularly *M. flabellata*–there was a heavy infestation of oysters growing on portions of their skeleton where there was no live tissue (either from the initial deployment or if tissue had receded *in situ*). In many cases, the oysters would have artificially inflated the weight of a coral and were therefore removed prior to measurement. At 1-year (February/March 2020), the tree branches and corals were once again brought into the seawater system tanks and reassessed as described above (Fig. 2D). Since the corals on the trees were similar in size to the initial mesocosm fragments and were deployed for 1 year in the field, their buoyant weights were compared to those obtained during the first year of the mesocosm study, thereby helping to ensure comparability between studies.

### Experiments and statistical analysis

Net growth (final weight–initial weight) of dry weight was used as the metric for calcification and normalized to the initial skeletal weight yielding a rate of mg g^−1^ day^−1^ (milligrams of skeletal weight increase per gram of initial skeletal weight per day). In order to test the question of growth performance in a land-based nursery as predictive of expected growth in ocean nurseries (*ex situ *vs* in situ*), only the corals from the ambient system were used for analysis. For *M. capitata*, 17 genotypes were compared across both the mesocosms and trees (*N* = 17, *n* = 50 mesocosm ramets, *n* = 81 tree ramets), and for *M. flabellata* there were 13 genotypes common to both culture methods (*N* = 13, *n* = 41 mesocosm ramets, *n* = 48 tree ramets). See Table S1 in Supplemental Information. A two-way ANOVA with culture method (tanks *vs* trees) and genotype as fixed factors followed by a Tukey’s HSD *post hoc* was fit for each species independently.

For the temperature stress-hardening analysis, only corals from the trees were used with genotypes that were common to both ambient and high temperature preconditioning histories from the previous experiment (*M. capitata*: *N* = 8 genotypes, *n* = 43 ambient temperature ramets, *n* = 45 high temperature ramets; *M. flabellata*: *N* = 8 genotypes, *n* = 32 ambient ramets, *n* = 26 high temperature ramets). See Table S2 in Supplemental Information. For the group (population) response comparison, the one-year net growth was split into two six-month increments, pre-heat stress (Jan/Feb 2019–Aug/Sept 2019) and post-heat stress (Aug/Sept 2019–Feb/Mar 2020). A two-way ANOVA with time period (pre- and post-heat stress) and temperature preconditioning treatment as fixed factors followed by a Tukey’s HSD *post hoc* was fit for each species independently. To assess if there was a response by genotype (individual) and previous temperature exposure, net growth for the whole year (Jan/Feb 2019–Feb/Mar 2020) was analyzed. For each species separately, a two-way ANOVA with genotype and temperature preconditioning treatment as fixed factors followed by a Tukey’s HSD *post hoc* was fit to test differences in growth, and a chi-square test was used to determine if there were differences in survivorship between ambient and high temperature groups.

At the midpoint and final assessments, corals were considered to be in good condition if they had normal looking coloration (not pale/bleached), had not experienced 25% (or more) tissue loss, and had not suffered predation of more than 25%. If there was evidence of predation or partial/whole ramet mortality at either assessment, those corals were excluded only from the net growth measurement portion of the analysis. For the ambient *vs* high temperature survival comparison, corals that died while on the trees were included. A between-species comparison of the overall number of corals surviving and remaining in good condition after a year on the trees was also examined with a chi square test. The calcification rate data for *M. capitata* culture method and temperature preconditioning were square root transformed to satisfy ANOVA assumptions; no data transformations were needed for *M. flabellata*. Normality assumptions were analyzed *via* diagnostic plots of the residuals and confirmed with a Shapiro-Wilk’s test. R version 3.5.3 (R Core Team, 2019) was used for ANOVAs and *post hoc* analyses, and chi-squares tests and graphics were made with GraphPad Prism 9 software (version 9.0.1; San Diego, CA).

## Results

### Mesocosm *vs* coral tree growth

**Figure 4:**
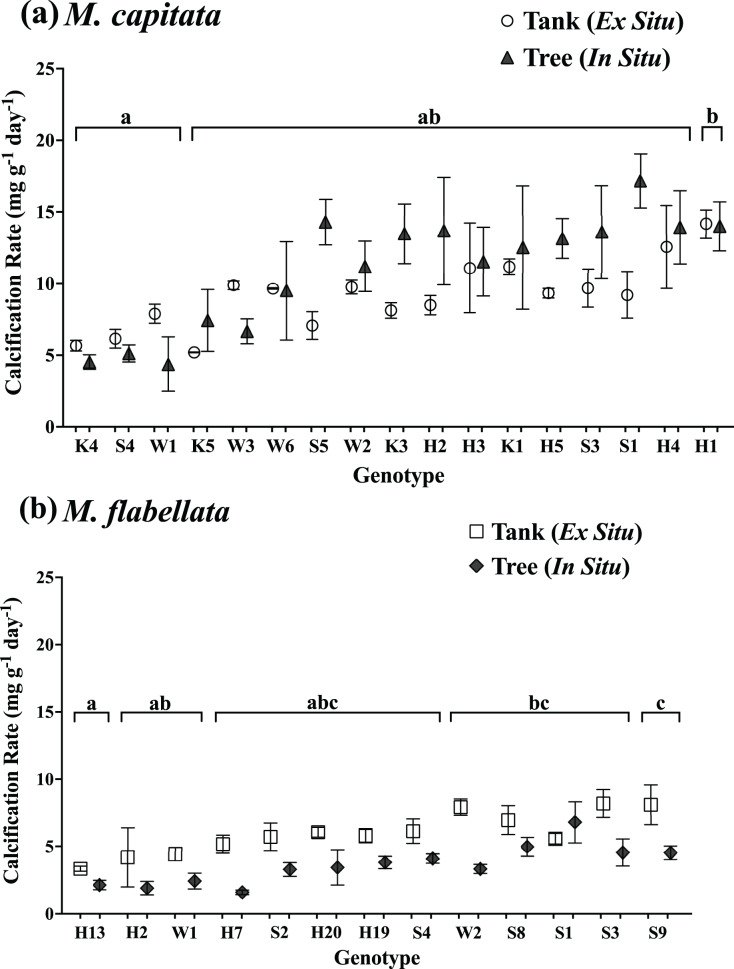
Calcification rate of corals by genotype and culture method. Comparison of calcification rate by coral genotype and method (tanks *vs* trees) after 1 year for (A) *M. capitata*: only genotype main effects were significant (*p* = 0.0003). (B) *M. flabellata*: the interaction of culture method and genotype was not significant (*p* = 0.0930), but the main effects of both genotype and method (*p* < 0.0001 for both) were. Variation in growth on the trees *vs* in mesocosms is explained primarily by genotype rather than culture method for *M. capitata*. For *M. flabellata*, growth was significantly impacted by culture method and genotype. Error bars are SE, and different letters above groups indicate significant difference in overall calcification genotypes.

**Figure 5:**
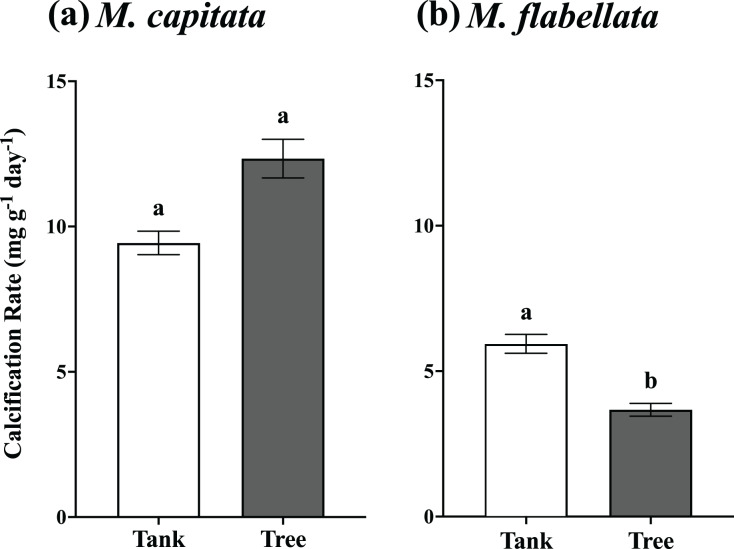
One year mean calcification rate for all fragments, tanks *vs* trees. (A) *M. capitata*: Calcification rate of all fragments on the trees is not significantly greater than tanks after 1 year but is trending toward significance (*p* = 0.088). (B) *M. flabellata*: Calcification rate of all fragments in the tanks is greater than on the trees after 1 year (*p* < 0.0001). The trees demonstrated marginally higher growth for *M. capitata*, while calcification rate for *M. flabellata* declined once on the trees. Error bars are SE, and different letters indicate significant difference.

The buoyant weight of *M. capitata* and *M. flabellata* corals previously grown in mesocosms and then moved to a coral tree nursery was tracked in both systems. Differences in calcification rate for *M. capitata* were attributed primarily to genotype (F_(16,97)_ = 3.1; *p* = 0.0003) rather than culture method. Neither effect of culture method (F_(1,97)_ = 2.97; *p* = 0.09) nor the interaction of genotype with method (F_(16,97)_ = 0.99; *p* = 0.476) were significant. A Tukey’s HSD *post hoc* test identified variation in growth among the genotypes (Fig. 4A; see Table S3 in Supplemental Information for ANOVA tables). When all the genotypes were combined, mean calcification rate on the trees for *M. capitata* (12.34 +/− 0.66 mg g^−1^ day^−1^) was trending greater than in the tanks (9.44 +/− 0.4 mg g^−1^ day^−1^), but it was not significantly greater (*p* = 0.09). See Table 1 and Fig. 5A.

**Table 1:** Growth (calcification rate) results of both species by culture method after 1 year in tanks (*ex situ*) *vs* on coral trees (*in situ*). The number of genotypes (*N*) and total number of pooled ramets (*n*) used for each analysis are provided.

		*M. capitata*		*M. flabellata*	
		Tanks	Trees	Tanks	Trees
Culture method	Genotypes (*N*) Pooled ramets (*n*)	*N* = 17 *n* = 50	*N* = 17 *n* = 81	*N* = 13 *n* = 41	*N* = 13 *n* = 48
	Calc. Rate (mg g^−1^ day^−1^) Mean ± SEM	9.43 ± 0.404	12.34 ± 0.664	5.95 ± 0.308	3.72 ± 0.227

For *M. flabellata*, both main effects, genotype (F_(12,63)_ = 4.26; *p* < 0.0001) and culture method (F_(1,63)_ = 61.1; *p* < 0.0001), were significant, but the interaction of genotype with method was not (F_(12,63)_ = 1.68; *p* = 0.093). The *post hoc* analysis also identified significant differences in performance among various genotypes (Fig. 4B; see Table S3 Supplemental Information for ANOVA tables). When the genotypes were combined for *M. flabellata*, the corals grew better in the mesocosms after one year; mean calcification rate was greater in the tanks (5.95 +/− 0.31 mg g^−1^ day^−1^) than on the trees (3.72 +/− 0.23 mg g^−1^ day^−1^; *p* < 0.0001). See Table 1 and Fig. 5B (and Table S3 in Supplemental Information for ANOVA tables). For *M. capitata*, growth on the trees was relatively consistent with growth in the tanks, with variation explained mostly through genotype, whereas the growth of *M. flabellata* was significantly impacted by culture method as well as genotype.

### Performance of outplanted, stress-hardened corals during a natural bleaching event

The maximum level of heat stress experienced by the corals in both experiments was relatively similar, reaching a peak of about 20–23 degree heating weeks (DHW, °C-weeks) in both the mesocosms and on the reef (Fig. 3A; Fig. S1 in Supplemental Information). While bleaching was observed in corals throughout the bay and on the HIMB reef where the trees were located during the course of this study, no *M. capitata* bleached and only one genotype of *M. flabellata* (Haleiwa-13) from the ambient temperature treatment history group visually paled in coloration. For both species, preconditioning the corals to high temperatures had no significant effect on growth in either species either before or after the natural bleaching event.

**Figure 6:**
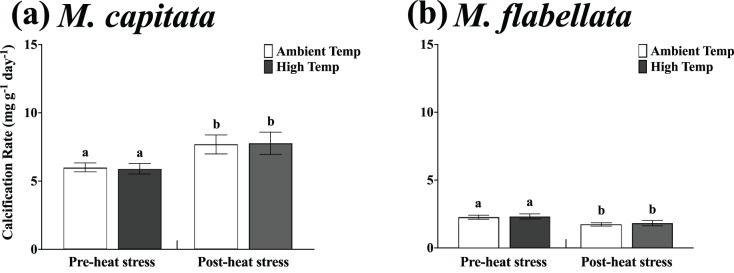
Calcification rate of ambient *vs* high temperature preconditioned corals on trees, pre- and post-heat stress. Mean growth rate of each group of ambient and high temperature preconditioned corals on the trees in six-month increments (before and after a natural warming event) for (A) *M. capitata* and (B) *M. flabellata*. For both species there was no difference in growth between ambient or high temperature preconditioned populations for either time period; temperature preconditioning treatment while in the mesocosms did not benefit growth during an *ex situ* warming event (*M. capitata*: *p* = 0.792; *M. flabellata*: *p* = 0.604). The calcification rate of both groups of *M. capitata* was greater post-heat stress (*p* = 0.04) while the reverse was true for *M. flabellata* (*p* = 0.002). Error bars are SE, and different letters indicate significant difference.

Specifically, the mean calcification rate of ambient and high temperature *M. capitata* corals on the trees from Jan-2019 to Aug-2019 (pre-heat stress) was 5.99 +/− 0.34 mg g^−1^ day^−1^ and 5.89 +/− 0.398 mg g^−1^ day^−1^, respectively. For the post-heat stress period (Aug-2019 to Feb-2020), the mean ambient and high temperature calcification rates were also not significantly different at 7.69 +/− 0.69 mg g^−1^ day^−1^ and 7.77 +/− 0.81 mg g^−1^ day^−1^, respectively (Table 2; Fig. 6A). A two-way ANOVA determined that there was no interaction of preconditioning with the pre- and post-heat stress time periods (F_(1,172)_ = 0.02; *p* = 0.89), and ambient *vs* high temperature (preconditioning) treatment was not a significant factor (F_(1,172)_ = 0.07; *p* = 0.79). The main effect for time period on the trees was significant, though near the threshold (F_(1,172)_ = 4.29; *p* = 0.04), with calcification rate for both groups greater during the post-heat stress time period.

**Table 2:** Growth (calcification rate) and survival of temperature preconditioning groups while on coral trees (*in situ* only) pre- and post-heat stress. The number of genotypes (*N*) and total number of pooled ramets (*n*) used for each analysis are provided.

		*M. capitata*		*M. flabellata*	
		Ambient temp	High temp	Ambient temp	High temp
Temperature group: Growth	Genotypes (*N*) Pooled ramets (*n*)	*N* = 8 *n* = 43	*N* = 8 *n* = 45	*N* = 8 *n* = 32	*N* = 8 *n* = 26
	Calc. Rate (mg g^−1^ day^−1^) Mean ± SEM, Pre-heat stress	5.99 ± 0.341	5.89 ± 0.398	2.26 ± 0.152	2.32 ± 0.191
	Calc. Rate (mg g^−1^ day^−1^) Mean ± SEM, Post-heat stress	7.69 ± 0.693	7.77 ± 0.814	1.71 ± 0.144	1.83 ± 0.195
Temperature group: Survival	Initial pooled ramets (*n*) Jan/Feb 2019	69	63	78	74
	Surviving pooled ramets (*n*) Aug/Sept 2019	62	59	70	65
	Surviving pooled ramets (*n*) Feb/Mar 2020	54	48	42	40

For *M. flabellata*, the pre- heat stress mean calcification rate was 2.26 +/− 0.15 mg g^−1^ day^−1^ for ambient corals and 2.32 +/− 0.19 mg g^−1^ day^−1^ for high temperature fragments. The mean calcification rate declined during the post-heat stress period to 1.71 +/− 0.14 mg g^−1^ day^−1^ and 1.83 +/− 0.19 mg g^−1^ day^−1^ for the ambient and high treatment groups, respectively (Table 2; Fig. 6B). The two-way ANOVA determined that there was no interaction between preconditioning with the pre- and post-heat stress time periods (F_(1,112)_ = 0.03; *p* = 0.856), and ambient *versus* high temperature (preconditioning) treatment, again, was also not a significant factor (F_(1,112)_ = 0.27; *p* = 0.604). Time period on the trees, however, was significant (F_(1,112)_ = 9.63; *p* = 0.002) indicating that the calcification rate for both groups significantly declined during the second six month, post-heat stress period compared to the first. For both species, growth of fragments was not influenced by previous temperature preconditioning treatment. The rate of calcification for both ambient and stress-hardened *M. capitata* increased post-heat stress, whereas *M. flabellata* had reduced calcification post-heat stress regardless of preconditioning, suggesting other factors impacted growth equally across treatments (Fig. 6). See Table S4 in Supplemental Information for two-way ANOVA details for both species.

**Figure 7:**
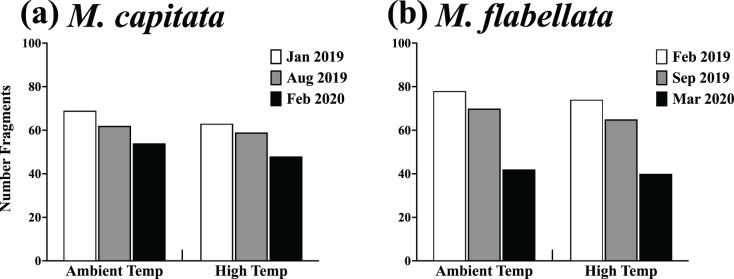
Survivorship of ambient and high temperature preconditioned corals on trees. Results of a chi-square analysis of surviving fragments from ambient and high temperature preconditioned groups for both (A) *M. capitata* (*X*^2^_(2,355)_ = 0.066, *p* = 0.967) and (B) *M. flabellata X*^2^_(2,369)_ = 0.011, *p* = 0.994. For both species there was no difference in survivorship between ambient and high temperature preconditioned corals while on trees after a warming event. The exposure to increased *ex situ* temperatures while in the mesocosms did not appear to benefit the fragments of either species *in situ* during a time of elevated temperatures while on the coral trees.

There was also no difference in post-heat stress survival between preconditioning groups of either species. Specifically, a chi-square analysis for both *M. capitata* (Table 1; Fig. 7A, *X*^2^_(2,355)_ = 0.066, *p* = 0.967) and *M. flabellata* (Table 1; Fig. 7B, *X*^2^_(2,369)_ = 0.011, *p* = 0.994) determined that, for both species, there was no difference in survivorship between ambient and stress-hardened corals while on trees, despite the prior temperature conditions in the mesocosms and similar warming in the fall of 2019 (Fig. 3A; Fig. S1).

**Figure 8:**
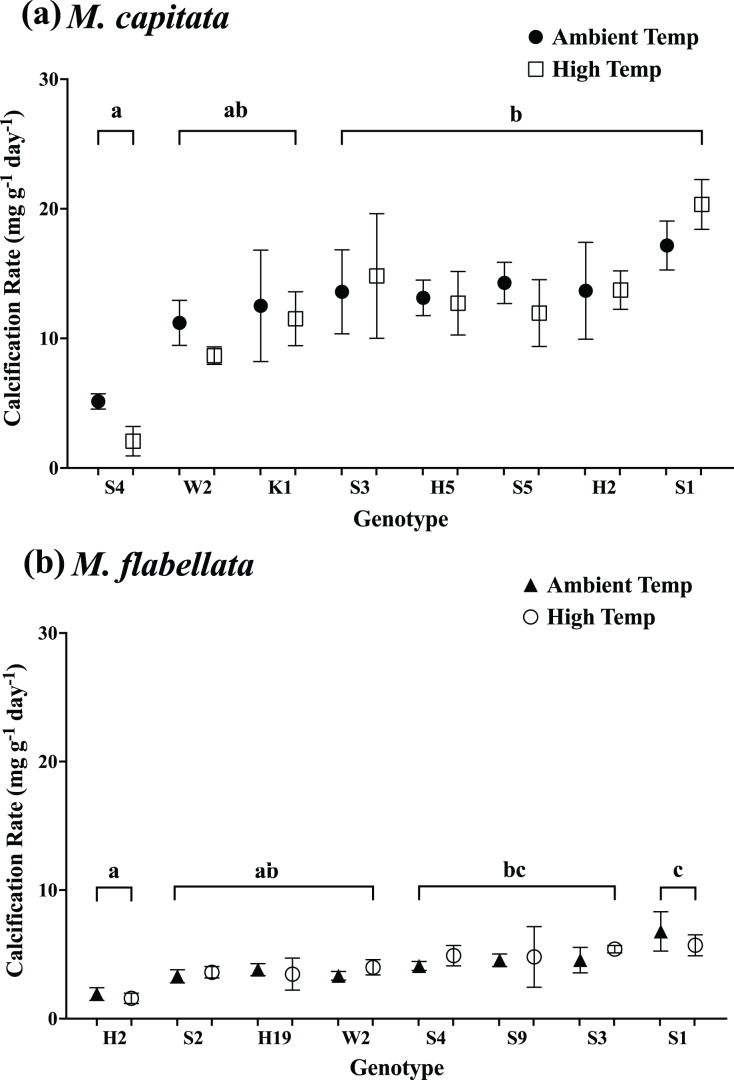
Calcification rate of corals by genotype and previous temperature treatment. Calcification rate by coral genotype and temperature preconditioning group (ambient *vs* high temperature) after 1 year while on trees during a natural warming event. For both species, only genotype main effects were significant (A) *M. capitata* (*p* = 0.0004) and (B) *M. flabellata* (*p* = 0.0002). Temperature preconditioning main effects were not significant (*M. capitata p* = 0.82; *M. flabellata p* = 0.49), and there was no significant interaction between genotype and temperature group (*p* = 0.92 and *p* = 0.91, respectively). For both species, exposure to elevated temperatures while in mesocosms did not benefit growth during a heat stress event in the ocean nursery. Variation in growth is explained by genotype but not previous temperature stress-hardening exposure. Error bars are SE, and different letters indicate significant difference among genotypes.

Additionally, for both species, when temperature preconditioning treatment was examined by genotype, the differences in growth between the ambient and high temperature corals were explained by genotype rather than temperature group. For *M. capitata*, neither the interaction of genotype with preconditioning temperature (F_(7,72)_ = 0.36; *p* = 0.92) nor the temperature group main effects (F_(1,72)_ = 0.05; *p* = 0.82) were significant, but genotype main effects were significant (F_(7,72)_ = 4.44; *p* = 0.0004); a Tukey’s *post hoc* comparison identified a significant difference between genotypes (Fig. 8A; see Table S5 in Supplemental Information for ANOVA tables). Similarly, for *M. flabellata* there was no interaction of genotype with temperature preconditioning (F_(7,42)_ = 0.39; *p* = 0.91), preconditioning main effects were not significant (F_(1,42)_ = 0.48; *p* = 0.49), but genotype main effects were significant (F_(7,42)_ = 5.4; *p* = 0.0002). The Tukey’s *post hoc* test found a significant difference between genotypes relative to the others (Fig. 8B; see Table S5 in Supplemental Information for ANOVA tables). For both species, stress-hardening had no effect on growth during a warming event *in situ*; the differences in growth were based upon genotype differences.

### General coral tree performance

Finally, there were far more *M. flabellata* than *M. capitata* ramets lost or damaged during these experiments. To examine this, we compared *M. flabellata* initial and final coral numbers remaining and in good condition to that of *M. capitata*. Corals were not considered in good condition if, during the year on the tree, they experienced any significant tissue loss or signs of predation (more than ~25% for either) or if they had died or were lost from the tree. At the initial deployment, all *M. capitata* (*n* = 216) and *M. flabellata* (*n* = 290) ramets were in good condition. After 1 year, 61% of *M. capitata* corals had survived, with 39% lost or considered not in good condition. By comparison, 14% of *M. flabellata* had survived, with a significantly greater 86% of ramets that were lost or in poor condition (*X*^2^_(1,679)_ = 60.33, *p* < 0.0001).

## Discussion

The devastation to and decline of reefs in the recent past (De’ath et al., 2012; Gardner et al., 2003; Pandolfi & Jackson, 2006) has spurred many in the field of marine conservation to shift focus from habitat protection (more passive, classic conservation biology) to more active intervention management or restoration ecology (Anthony et al., 2017; Epstein, Bak & Rinkevich, 2003; van Oppen et al., 2017). This shift has fueled the development of a number of novel strategies, with variations, that exist under the umbrella of conservation and restoration, including land-based aquaria and ocean-based nurseries that utilize fragments (asexual reproduction) and larval recruits (sexual reproduction) as strategies for restoration (Chamberland et al., 2015; dela Cruz & Harrison, 2017; Forsman et al., 2018; Guest et al., 2014; Nedimyer, Gaines & Roach, 2011; Page, Muller & Vaughan, 2018; Rinkevich, 2000; Ware et al., 2020). The concept of ‘coral gardening’ within an ocean nursery has become a familiar method used in restoration projects that has shown promise, and the overwhelming majority of corals used in nursery restoration have typically been fast growing, branching species (Boström-Einarsson et al., 2020; Forsman et al., 2012; Rinkevich, 2014).

One of the initial measures of a successful restoration program is not only survival of the outplanted corals but also rapid growth after relocation to the ocean (Boström-Einarsson et al., 2020). It is reasonable for a culture facility to want to target those individual genotypes that have demonstrated previous success. However, the confines of a maintained *ex situ* culture facility are substantially different from the selective pressures in the open ocean, and growth in aquaria or among time points might not translate to success on the reef (Edmunds & Putnam, 2020). The present study examined genotype performance, survivorship, and the effectiveness of stress-hardening *in situ* of *M. capitata* and *M. flabellata* corals that had known growth histories from an *ex situ* mesocosm nursery. These experiments addressed questions surrounding reef restoration, specifically: (1) is previous growth in an *ex situ* mesocosm system a predictor of growth in an *in situ* ocean nursery; and (2) does preconditioning corals to thermal stress in an *ex situ* culture facility benefit them *in situ* during a natural bleaching event?

Our results indicate that variation in growth was better explained through genotype than culture method for one of the species (*M. capitata*, Fig. 4A), and this was consistent with other studies that have found genotype to be a predictor of growth success in a nursery setting (Kuffner et al., 2017; Lirman et al., 2014; Lohr & Patterson, 2017; Morikawa & Palumbi, 2019; O’Donnell et al., 2017) and in a reciprocal outplant study (Drury, Manzello & Lirman, 2017). This expected result for *M. capitata* was tempered somewhat by genotype and culture method both providing variation in growth for *M. flabellata*, which complicates overall predictions (Fig. 4B). In general, if a colony of *M. capitata* grew well in our mesocosm nursery, it could be expected to perform similarly well when moved to a coral tree ocean nursery. These growth rates were also about three times higher than those reported in previous mesocosm studies, suggesting that differences in *ex situ* culture conditions such as water flow (Jokiel et al., 2008) and irradiance (Jury & Toonen, 2019) can dramatically affect calcification rates. Although some genotypes of *M. flabellata* grew well in both a mesocosm setting and an ocean nursery, a different nursery design (other than coral trees) that accommodates the encrusting growth formation could result in more comparable growth rates between *ex situ* and *in situ* methods.

There is some optimism that individual acclimatization or population adaptation of corals to rising ocean temperatures will drive reef resilience to climate change (Coles et al., 2018; Majerova et al., 2021; Middlebrook, Hoegh-Guldberg & Leggat, 2008; Thomas et al., 2018), and while there are probable limits (Ainsworth et al., 2016; Hughes et al., 2017), various intervention strategies are attempting to direct that effort (Hagedorn et al., 2021; Hancock et al., 2021; van Oppen et al., 2017; van Oppen et al., 2015). Because Kāne‘ohe Bay again experienced elevated temperatures and subsequent bleaching throughout the bay and around HIMB (where the nursery trees were located) during the summer and fall of 2019, we examined the response between individual and groups of corals with and without a history of previous exposure to elevated temperatures.

For both species during a warming event while on the trees, the preconditioning to heat stress (simulated stress events) did not have any beneficial impact on calcification rate or survival compared to those previously maintained in ambient mesocosm temperatures. Corals throughout the bay, including colonies near the section of reef where the coral trees were located, visually bleached; there was no paling or bleaching of *M. capitata* on the trees, and only one genotype of *M. flabellata* bleached. However, for both ambient and high temperature preconditioned groups of *M. capitata*, the calcification rate was greater in the six-month post-bleaching event period than prior (Fig. 6A). The opposite was true for *M. flabellata*, where growth was reduced in both groups after bleaching compared to the initial period (Fig. 6B). In addition, temperature treatment had no impact on growth of individual genotypes for both species (Fig. 8). The lack of preconditioning benefit was true at the population level (treatment group) as well as at the individual level (genotype) for both species despite similar level and duration of heat stress (Fig. 3A; Fig. S1). It is possible that these results are due to increased heterotrophic feeding by *M. capitata* under thermal stress (Grottoli, Rodrigues & Palardy, 2006) while the coral tree design and location were not optimal for *M. flabellata*.

Similarly, Middlebrook et al. (2012) found *Acropora millepora* colonies that were experimentally preconditioned to warmer temperatures were not more bleaching tolerant during the following bleaching event (though their symbionts did show improvement), yet the reverse was true for *A. aspera* (Middlebrook, Hoegh-Guldberg & Leggat, 2008); some Caribbean species have shown mixed results to repeated warming events (Grottoli et al., 2014). While the preconditioning of *Pocillopora acuta* (formerly identified as *P. damicornis*) has conveyed thermal tolerance to larvae *via* transgenerational acclimatization (Putnam & Gates, 2015), that effect was lost among the adults (Jury, Delano & Toonen, 2019). Conversely, Morikawa & Palumbi (2019) were able to construct a multispecies nursery that experienced less bleaching during a warming event by identifying specific colonies that were thermally tolerant in naturally-occurring areas of the reef, similar to our finding that genotype was the predominant significant factor in determining coral responses to these experiments. While individual genotypes of some species might be resilient to repeated warming, experimentally subjecting corals to simulated stress events in order to stress harden them to future warming or to select those individuals most likely to be “winners” appears not to be a practical strategy. This conclusion is corroborated by Barott et al. (2021) who likewise recently found no alteration of bleaching response among preconditioned corals in a reciprocal transplant experiment in Kāne‘ohe Bay. Taken together, thermally preconditioning or stress hardening corals would likely also not transfer to future generations.

As previously stated, the calcification rate for *M. capitata* was greater on the trees than in the mesocosms (though the difference was not statistically significant), while the reverse was true for *M. flabellata*, and there were far more *M. flabellata* lost while on the trees. The overall better success on the trees for *M. capitata* might be explained by the growth formation, natural history of the two species, and tree location in the bay. With its branching and plating morphology, *M. capitata* is a common, wide-ranging species found across variable reef habitats and depths; *M. flabellata*, however, is an encrusting species restricted to more narrow bands of reefs with high wave action and irradiance (Fenner, 2005; Hunter & Evans, 1995; Rodgers et al., 2015). To date, the most commonly used corals in restoration projects have been those with branching morphologies (Boström-Einarsson et al., 2020). Additionally, the permitted location for the coral trees had abundant wild *M. capitata* colonies but was not an ideal habitat for *M. flabellata* (Bahr, Jokiel & Toonen, 2015; Hunter & Evans, 1995; Richards Donà, 2019).

Within a few weeks of deployment, the coral trees (PVC frame, fiberglass branches, rope anchors) were inundated with filter feeding organisms (oysters, tunicates, sponges) rather than filamentous algae, and these organisms, in particular the oysters, would additionally recruit onto areas of the coral that did not have live tissue. Unless a portion of the fragment died while on the tree, this was typically not an issue for *M. capitata*. For *M. flabellata*, however, its encrusting morphology allowed the underside of the fragment to be exposed, and there was substantial recruitment of filter feeders, primarily oysters, while hanging from the branches. Unlike *M. capitata*, this unnatural exposure of the underside appeared to lead to heavy predation on the fragments of *M. flabellata* throughout the study–large sections of fragments missing and even the shearing of monofilament line whereupon whole fragments disappeared from the trees. It is likely, then, that the poor performance of *M. flabellata* on the trees was due to a combination of less-than-ideal habitat location and ill-suited morphology for the coral tree design that led to an increased likelihood of predation. While fish predation has also been identified as an obstacle for massive corals in other restoration efforts (Koval et al., 2020), *M. flabellata* might have better success in a nursery location near a more oceanic setting and utilizing the flat plate design for the coral tree branches rather than the line suspension method.

This study examined whether growth in an *ex situ* nursery could accurately predict *in situ* growth of fragments and whether there was a benefit of stress hardening corals prior to outplanting. We focused on growth and survival of corals in a coral tree ocean nursery and the role of genotype in nursery growth; however, there are more considerations for a nursery and restoration than growth alone. The Caribbean staghorn coral, *Acropora cervicornis*, is one of the most intensely studied species in restoration projects. Examinations of nursery and restored *A. cervicornis* have suggested the fastest growing genotypes of restored colonies might trade growth for thermal stress recovery (Ladd et al., 2017), a potential tradeoff of skeletal density with branch extension (Lohr & Patterson, 2017), and growth alone when moved from nursery to reef was not predictive of success (O’Donnell et al., 2018). Additionally, based on the outcome of this study and (Barott et al., 2021) the time, effort, and increased expense of thermal preconditioning to promote stress hardening is not warranted.

In a large, scaled-up restoration program, it may be cost prohibitive or logistically infeasible to track multiple metrics of every coral in the system. If restoration continues to be employed as a strategy for reef conservation, there is a need for rapid, low-cost and low-tech proxies for potential success to keep costs manageable (Guest et al., 2014; Morikawa & Palumbi, 2019; Young, Schopmeyer & Lirman, 2012), accompanied by more accurate and standardized metrics of growth and performance (Edmunds & Putnam, 2020). Baums et al. (2019) note that in addition to growth rate and genetic diversity, disease resistance, fecundity, partial tissue loss, rate of wound healing, symbiont variability, bleaching resistance/resilience, and other traits are all factors to consider. Reducing stressors must continue before significant population growth and recovery will occur (Ware et al., 2020). Even if fast-growing, acclimatized, or more thermally tolerant corals can be used in meaningful, large-scale successful restoration projects, such efforts will not justify complacency toward climate change mitigation.

## Supplemental Information

10.7717/peerj.13112/supp-1 Supplemental Information 1 Degree Heating Weeks, Tanks *vs* Trees. Thermal stress experienced by corals in the high temperature mesocosms (*ex situ* tanks) and all corals while on the *in situ*coral trees expressed in degree heating weeks (DHW). Temperature stress reached a peak of approximately 20–23 DHW in both experiments. DHW are calculated using the mean monthly maximum (MMM) temperature baseline of 26.98 **°**C for the Main Hawaiian Islands from NOAA Coral Reef Watch with a nominal bleaching threshold of MMM+1 **°**C, per the methodology found in Skirving et al. (2020).

10.7717/peerj.13112/supp-2 Supplemental Information 2 Raw data for normalized net coral growth.

10.7717/peerj.13112/supp-3 Supplemental Information 3 Supplemental Tables.

## Additional Information and Declarations

### Data Availability

The following information was supplied regarding data availability: The raw data is available in the Supplemental File.
