# Supplementary material for: Growth and survival among Hawaiian corals outplanted from tanks to an ocean nursery are driven by individual genotype and species differences rather than preconditioning to thermal stress
Source: PeerJ. 2022 Mar 23;10:e13112. doi: 10.7717/peerj.13112 (PMC8957268; doi:10.7717/peerj.13112)
Supplement: Supplemental Information 3 [file peerj-10-13112-s003.pdf]

Supplementary Information for

Growth and survival among Hawaiian corals outplanted from tanks to an ocean nursery are driven by individual genotype and species differences rather than preconditioning to thermal stress

E.M. Henley, J. Bouwmeester, C.P. Jury, R.J. Toonen, M. Quinn, C.V.A. Lager, and M. Hagedorn

Send correspondence to: henleym@si.edu or emhenley@hawaii.edu

This file includes:
Figure S1: Degree Heating Week (DHW) comparison of high temperature tanks and coral trees
Tables S1 to S5: coral identification used in analyses and results of two-way ANOVAs

Additional supplementary information for this manuscript:
"Supp_Data_S1" is an Excel file that includes the data for this manuscript. Each tab in the Excel file corresponds to the normalized net growth (mg g^{-1} day^{-1}) data for corals used in each analysis.

1. Genotype and culture method (tanks vs. coral trees) growth after one year – *M. capitata*
2. Genotype and culture method (tanks vs. coral trees) growth after one year – *M. flabellata*
3. Genotype, temperature preconditioning history while in the tanks (ambient vs. high), and time period of growth measurement while on the trees (first six months pre-bleaching, second six months post-bleaching, and the full one year on trees) – *M. capitata*
4. Same as #3 above but for *M. flabellata*
5. Temperature data (°C) for both the tanks and coral trees

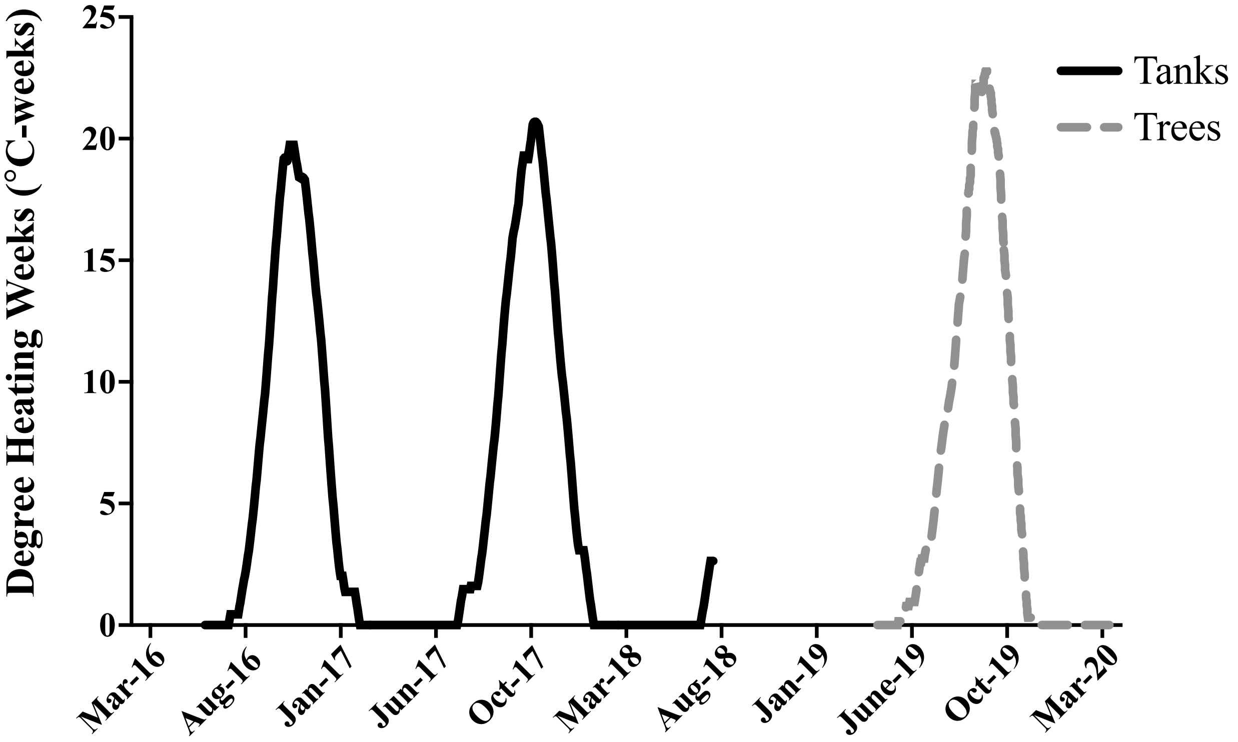

Figure S1. Degree Heating Weeks, Tanks vs. Trees.
Thermal stress experienced by corals in the high temperature mesocosms (*ex situ* tanks) and all corals while on the *in situ* coral trees expressed in degree heating weeks (DHW). Temperature stress reached a peak of approximately 20-23 DHW in both experiments. DHW are calculated using the mean monthly maximum (MMM) temperature baseline of 26.98 °C for the Main Hawaiian Islands from NOAA Coral Reef Watch with a nominal bleaching threshold of MMM+1 °C, per the methodology found in Skirving et al. (2020).

Table S1. Identification of genotypes and ramets per species for culture method, tanks (*ex situ*) vs. coral trees (*in situ*) using corals from the ambient temperature mesocosms only.

M. capitata **Culture Method**		
Genotype	Tank Ramets	Tree Ramets
HIMB1	6	8
HIMB2	6	4
HIMB3	2	5
HIMB4	2	5
HIMB5	2	8
Kahe1	2	5
Kahe3	2	5
Kahe4	2	2
Kahe5	2	3
Sampan1	2	8
Sampan3	2	6
Sampan4	2	2
Sampan5	2	8
Waimanalo1	6	2
Waimanalo2	6	2
Waimanalo3	2	4
Waimanalo6	2	4

M. flabellata **Culture Method**		
Genotype	Tank Ramets	Tree Ramets
Haleiwa2	2	2
Haleiwa7	2	2
Haleiwa13	2	4
Haleiwa19	2	5
Haleiwa20	2	3
Sampan1	5	2
Sampan2	6	4
Sampan3	2	3
Sampan4	2	4
Sampan8	2	5
Sampan9	2	6
Waimanalo1	6	2
Waimanalo2	6	6

Table S2. Identification of genotypes and ramets per species for temperature preconditioning, using the coral tree (*in situ*) fragments with a previous history of ambient vs. high temperature preconditioning.

***M. capitata* Temp Preconditioning**		
Genotype	Ambient Temp Ramets	High Temp Ramets
HIMB2	4	9
HIMB5	8	4
Kahe1	5	4
Sampan1	8	9
Sampan3	6	7
Sampan4	2	2
Sampan5	8	5
Waimanalo2	2	5

***M. flabellata* Temp Preconditioning**		
Genotype	Ambient Temp Ramets	High Temp Ramets
Haleiwa2	2	3
Haleiwa19	5	2
Sampan1	2	3
Sampan2	4	5
Sampan3	3	2
Sampan4	4	5
Sampan9	6	2
Waimanalo2	6	4

Table S3. Two-way ANOVA results for culture method (*ex situ* = tanks, *in situ* = coral tree) and genotype, *M. capitata* and *M. flabellata*.

M. capitata

Factor	Sum Sq	Df	F value	Pr(>F)
Genotype	21.8	16	3.1	0.0003 ***
Method	1.31	1	2.97	0.088 .
Genotype:Method	6.94	16	0.988	0.476
Residuals	42.58	97		

M. flabellata

Factor	Sum Sq	Df	F value	Pr(>F)
Genotype	103.77	12	4.26	6.569e-05 ***
Method	123.97	1	61.14	7.388e-11 ***
Genotype:Method	40.87	12	1.68	0.093 .
Residuals	127.74	63		

Table S4. Two-way ANOVA results for ambient vs. high temperature preconditioning (Temp) population of fragments for pre- and post-heat stress (Time Period) while on coral trees (*in situ*), *M. capitata* and *M. flabellata*.

M. capitata

Factor	Sum Sq	Df	F value	Pr(>F)
Temp	0.042	1	0.0698	0.7919
Time Period	2.57	1	4.287	0.0399 *
Temp:Time Per.	0.011	1	0.019	0.8907
Residuals	103.26	172		

M. flabellata

Factor	Sum Sq	Df	F value	Pr(>F)
Temp	0.221	1	0.2699	0.604
Time Period	7.89	1	9.631	0.0024 **
Temp:Time Per.	0.027	1	0.033	0.8556
Residuals	91.8	112		

Table S5. Two-way ANOVA results for ambient vs. high temperature preconditioning (Temp) fragments and genotype while on coral trees (*in situ*), *M. capitata* and *M. flabellata*.

M. capitata

Factor	Sum Sq	Df	F value	Pr(>F)
Genotype	23.6	7	4.44	0.000381***
Temp	0.039	1	0.05	0.821311
Genotype: Temp	1.937	7	0.36	0.920165
Residuals	54.72	72		

M. flabellata

Factor	Sum Sq	Df	F value	Pr(>F)
Genotype	63.65	7	5.405	0.00018 ***
Temp	0.801	1	0.476	0.49398
Genotype: Temp	4.55	7	0.386	0.90516
Residuals	70.66	42		

References

Skirving W, Marsh B, De La Cour J, Liu G, Harris A, Maturi E, Geiger E, and Eakin CM. 2020. CoralTemp and the Coral Reef Watch Coral Bleaching Heat Stress Product Suite Version 3.1. *Remote Sensing* 12:3856.
